# Adverse event reporting in healthcare—Regulatory frameworks, institutional governance, and economic implications with evidence from Poland

**DOI:** 10.3389/fpubh.2026.1891735

**Published:** 2026-10-06

**Authors:** Jolanta Agnieszka Pacian, Marlena Jolanta Piekut

**Affiliations:** 1 Department of Medical and Pharmaceutical Law, Department of Psychosocial Aspects of Medicine, Medical University of Lublin, Lublin, Poland; 2 College of Economics and Social Sciences, Warsaw University of Technology, Warsaw, Poland

**Keywords:** adverse event reporting, health economics, health law, patient safety management, Poland

## Abstract

Adverse events constitute a major challenge not only for patient safety but also for the effectiveness, sustainability and equity of healthcare systems. The present analysis demonstrates that patient safety cannot be reduced to a purely clinical or legal issue, as adverse events generate substantial social and economic consequences that frequently extend beyond healthcare institutions and are borne by patients and their households. The findings indicate that the current Polish model of adverse event monitoring, based predominantly on internal reporting mechanisms within individual healthcare entities, provides limited opportunities for national learning, regulatory evaluation and evidence-based policy-making. The absence of a centralized adverse event register restricts the ability to assess the effectiveness of patient safety interventions, estimate the full social and economic burden of adverse events and identify systemic risk factors that require regulatory action. Effective reporting systems require an organizational culture based on trust, transparency and learning, supported by the principles of no-blame and just culture, which encourage incident reporting while maintaining accountability for reckless or intentional misconduct. Therefore, future reforms should focus on strengthening information infrastructure, improving data quality and comparability, and developing integrated national reporting systems that facilitate organizational learning and regulatory evaluation. The establishment of a central adverse event register, combined with transparent reporting mechanisms and systematic assessment of patient safety policies, could significantly improve both patient outcomes and the allocative efficiency of healthcare systems. Integrating legal and economic perspectives into patient safety governance is essential for designing regulations that adequately reflect the full social costs of adverse events and promote more resilient, equitable and patient-centered healthcare systems.

## 1 Introduction

The aim of this article is to present the legal and economic conditions of adverse events occurring in healthcare entities, with particular emphasis on Poland. The article examines patient safety regulation from an integrated legal, institutional and health economics perspective, using Poland as an illustrative case within the broader international framework of patient safety governance.

The method used is a dogmatic-legal method consisting of an analysis of the applicable legal regulations and their systemic interpretation, supplemented by an analysis of the economic and institutional effects of the regulations. Specific criteria (content and quality) were applied when selecting the academic literature. These include: 1. Thematic relevance: the article directly addresses the functional questions and the defined objective. 2. Originality and scientific contribution: the publication presents groundbreaking conclusions and new methods. 3. Source credibility: articles from reputable peer-reviewed academic journals, books, and reports. This review follows a narrative, rather than a systematic, review methodology, consistent with the interpretive and doctrinal character of legal scholarship. Relevant sources were identified through targeted searches of Scopus, Web of Science, PubMed, LEX/el and Google Scholar, using combinations of the search terms “adverse event,” “patient safety,” “incident reporting system,” and “health economics,” together with jurisdiction-specific terms corresponding to the countries analyzed. These searches were supplemented by primary legal sources (statutes, regulations) and official institutional reports and publications identified through targeted searches of national health-authority, patient-ombudsman and other government websites. The search was not restricted to a fixed publication date range; however, priority was given to recent sources, particularly those published from the introduction of the Act of 16 June 2023 on Quality in Healthcare and Patient Safety onwards, reflecting the currency of the regulatory framework under discussion, while earlier foundational works in law and health economics were retained where relevant to the theoretical framework. Given the narrative character of the review, no formal inclusion/exclusion protocol or quality-appraisal tool was applied; sources were instead assessed against the criteria described above.

To present legal data from various countries—ensuring the representativeness of legal systems (model approaches)—member states representing traditional legal systems were selected. This approach allows for an examination of how different doctrinal systems monitor and record events. Countries were selected that are distinguished by their commitment to a “no-blame culture”—where the person reporting an incident need not fear negative consequences for disclosing it—as well as those where adverse event reporting systems are based on two fundamental principles: making risk visible and preventing harm.

The study addresses the following research question: how do legal frameworks governing adverse event reporting influence the effectiveness, transparency and economic performance of healthcare systems?

In connection with the research question, the following specific problems were posed:

1. What role does the Global Action Plan for Patient Safety 2021–2030 play in the monitoring of adverse events? (Specific problem 1 was discussed in the section titled-Global Action Plan for Improving Patient Safety 2021–2030).
2. How are adverse events defined and what is the significance of factors that increase the risk of their occurrence? (Specific problem 2 was discussed in the section titled-Defining adverse events and factors increasing the risk of their occurrence; Interventions for improving patient safety in healthcare systems).
3. What legal regulations apply to the internal quality management system and monitoring of adverse events in various countries, based on the representativeness of legal systems? (Specific problem 3 was discussed in the section titled-Management of adverse events in different countries).
4. Can the adverse event monitoring system be used as a tool to reduce the direct and indirect costs of preventable health damage and contribute to protecting patients' health capital, and if so, to what extent? (Specific problem 4 was discussed in the section titled-Factors affecting the risk of adverse events; Incidence and impact of adverse events).

The Conceptual Framework for the International Classification for Patient Safety was developed to standardize the terminology and categorization of adverse events, enabling data comparison between different healthcare systems. The ICPS defines, among other things, the types of incidents, their causes, circumstances, and potential consequences, thus providing a solid basis for error analysis and the implementation of corrective measures (1).

Against this international background, Poland introduced the Act of 16 June 2023 on Quality in Healthcare and Patient Safety, which established mandatory internal adverse event monitoring systems for healthcare providers (67). The Polish regulatory framework is analyzed in this article as a case study illustrating broader challenges related to patient safety governance and regulatory effectiveness.

Adverse events are consistently recognized at the international level as a problem with a clear economic dimension (2–4). However, despite this growing body of evidence, relatively little attention has been devoted to analyzing patient safety regulation through an integrated legal, institutional and economic perspective.

This gap provides the rationale for the present study, which examines how legal architecture, reporting mechanisms and institutional arrangements influence both patient safety and the broader economic consequences of adverse events.

## 2 Global action plan for improving patient safety 2021–2030

The Global Action Plan for Improving Patient Safety 2021–2030 provides a framework for individual countries to develop national patient safety strategies and to harmonize existing policy instruments aimed at improving patient safety across healthcare systems (70). The Plan contains seven strategic objectives and 35 implementation strategies.

The strategic objectives are: (1) policies to eliminate avoidable harm in healthcare; (2) high-reliability health systems; (3) safety of clinical processes; (4) patient and family engagement; (5) education and training of healthcare professionals; (6) information, research and risk management; and (7) synergy, partnership and solidarity (4).

The Action Plan recognizes patient safety as a core component of healthcare quality and encourages Member States to strengthen governance structures, reporting systems and organizational learning.

Accordingly, WHO recommends that Member States establish comprehensive patient safety frameworks, develop effective adverse event reporting mechanisms and integrate patient safety requirements into national legislation and healthcare policy (4).

The economic implications of adverse events and the efficiency of regulatory interventions are discussed later in the article in the context of health economics and the Polish regulatory framework.

## 3 Case study—Poland

### 3.1 Legal framework

In Poland, the legal definition of an adverse event is provided in Article 2(8) of the Act of 16 June 2023 on Quality in Healthcare and Patient Safety (5, item 1692). According to this provision, “*an adverse event is an event that occurs during the provision of, or as a result of the provision or failure to provide, healthcare, causing or likely to cause a negative effect on the health or life of a patient, in particular death, damage to health or health disorder, illness, life-threatening situation, the need for hospitalization or its prolongation, as well as bodily injury or health disorder of the fetus; an event whose effect is the expected effect of properly provided healthcare services does not constitute an adverse event.”*

The Polish definition provides a legal basis for mandatory reporting while distinguishing adverse events from expected outcomes of correctly delivered healthcare services.

Typical examples of adverse events include patient falls, medication errors, healthcare-associated infections, failure to obtain informed consent and delayed recognition of life-threatening conditions.

Although the fundamental concept of an adverse event is broadly consistent across Europe, reporting systems differ regarding which incidents are subject to mandatory notification. National systems may classify reportable events according to severity, event type or broader patient safety criteria, reflecting differences in regulatory priorities and institutional organization (5, 6).

In this context, the issue of legal liability for adverse events is also crucial. As a general rule, liability for an adverse event rests with: 1. the head of the medical entity (organizational liability)—who is responsible for implementing and managing the internal control and risk management system; 2. the medical entity (bears civil liability—specifically regarding compensation—for harm caused to a patient by medical personnel during the provision of services, based on either the risk principle or the fault principle, e.g., Articles 415 and 430 of the Civil Code)^1^; 3. as well as individuals practicing medical professions, such as doctors and nurses (who bear liability if an adverse event occurs due to their error, negligence, or failure to exercise due care) (7). This liability may take the form of civil liability (whether in tort—Article 415 of the Civil Code—or in contract—Article 470 of the Civil Code), professional liability (before professional self-regulatory bodies), and criminal liability (8). In the context of criminal liability, A. Liszewska defines a medical error as a violation of safety rules and professional conduct standards by a doctor (acting consciously) that infringes upon legally protected interests such as human life or health. The author asserts “that committing an error within the meaning of Article 9 § 2 of the Penal Code constitutes a failure to exercise the caution required under the given circumstances and may serve as the basis for holding a doctor criminally liable for committing an unintentional prohibited act” (72).

M. Filar lists the following prerequisites for the criminal liability of doctors: first, culpability; and second, the occurrence of an error—defined as an act contrary to medical knowledge and practice that results in a negative outcome, specifically the infringement of a legally protected interest or the exposure of such an interest to immediate danger (9). Third, establishing a causal link between the doctor's conduct and the adverse effect on the patient's health or life is an essential element. It should be noted that, in practice, both establishing this causal link and determining the degree and form of the doctor”s culpability for a medical error can present certain difficulties. As M. Filar further emphasizes, this means that

“it is essential for the doctor's conduct to be at least one of the causes without which the result would not have occurred. The involvement of other causes in the chain of causation does not absolve the doctors of criminal liability, provided they could have at least foreseen them” (9).

Patients who have suffered harm due to adverse medical events may pursue their claims through criminal or civil proceedings, or via the Medical Events Compensation Fund; the latter—pursuant to Art. 67zi of the Act of November 6, 2008, on Patient Rights and the Patient Ombudsman—is a state-run earmarked fund established to pay compensation benefits in the event of medical incidents (10). The Medical Events Compensation Fund is administered by the Patient Ombudsman. The Medical Events Compensation Fund provides a fast, out-of-court avenue for obtaining compensation for individuals who have suffered bodily injury, health impairment, or a hospital-acquired infection during a hospital stay. In the event of a patient's death, the benefit is also payable to their next of kin. The Fund covers medical events occurring in hospitals—including psychiatric and health resort facilities—in connection with the provision of publicly funded healthcare services (such as those guaranteed and financed by the National Health Fund) (11).

Importantly, obtaining the benefit does not require proving the fault of the medical entity. The fundamental basis for granting the benefit is the determination that a medical incident occurred which, with a high degree of probability, could have been avoided had the healthcare service been provided to the patient in accordance with current medical knowledge, or had a different available method of treatment or diagnosis been employed. K. Buczkowski and P. Bachmat aptly note that

“the Act of 6 November 2008 on Patient Rights and the Patient Ombudsman leaves the applicant with the ultimate choice of whether to accept the compensation benefit in the amount determined in the Patient Ombudsman's decision or to pursue compensation through a civil lawsuit” (12).

For, pursuant to Art. 67zc of the Act of 6 November 2008 on Patient Rights and the Patient Ombudsman, within 30 days of the date on which the decision granting the compensation benefit and determining its amount became final, the applicant must submit a statement to the Ombudsman either accepting or declining the compensation benefit. The Ombudsman informs the applicant of the requirement to submit such a statement, advising them of the consequences of both submitting and failing to submit it. Submitting a statement accepting the compensation benefit is tantamount to the applicant waiving any claims for damages, a pension, or monetary compensation for non-pecuniary loss arising from the medical incident, specifically regarding harm that became apparent by the date the application was submitted.

The amount of the compensation benefit arising from a single medical event for a single applicant is, in the case of: (1) infection with a biological pathogen—from PLN 2,000 to PLN 200,000; (2) bodily injury or impairment of health—from PLN 2,000 to PLN 200,000; (3) the death of a patient—from PLN 20,000 to PLN 100,000 (Article 67s—Act of 6 November 2008 on Patient Rights and the Patient Rights Ombudsman, 11). Furthermore, the amount of the compensation benefit for impairment of health may not exceed PLN 150,000, and the amount of the compensation benefit for the burden of treatment may not exceed PLN 75,000, as is also the case for the deterioration of quality of life.^2^

Operational data for 2025 show a substantial increase in activity compared with the Fund's first full year of operation. In 2023 (from 6 September, when applications could first be submitted) only 111 applications were filed. In 2024 the number rose to 1,574 applications, and in 2025 more than 2,500 applications were submitted to the Patient Ombudsman. In 2025 the Ombudsman issued more than 1,314 decisions, of which 586 granted compensation benefits totaling PLN 34 million (compared with approximately PLN 9.5 million paid out in 2024). The average benefit in 2025 amounted to approximately PLN 58,000. The awarded sums supported 352 persons in the process of recovery and 234 next of kin of deceased patients (13–15).

### 3.2 Institutional architecture

In Poland, healthcare entities providing publicly funded healthcare services are required to implement an internal quality management and adverse event monitoring system in accordance with the Act of 16 June 2023 on Quality in Healthcare and Patient Safety (5, item 1692). The introduction of such a system represents a form of regulatory intervention aimed at improving patient safety and strengthening the quality of healthcare services.

The system imposes new organizational responsibilities on healthcare providers and requires the allocation of human, organizational and financial resources to reporting, analyzing and preventing adverse events.

The implementation of monitoring systems generates direct costs related to staff training, documentation and reporting procedures, as well as indirect costs resulting from the additional administrative burden placed on healthcare personnel. This pattern is consistent with broader research on administrative burden in regulatory policy, which shows that newly introduced reporting and compliance obligations frequently generate implementation costs disproportionate to their original policy design (16).

The current Polish model is based primarily on internal monitoring systems operating at the level of individual healthcare entities. A recent survey-based report published by the Polish Patient Ombudsman (Rzecznik Praw Pacjenta) in September 2025—the first analysis of its kind in Poland—confirms this fragmented picture empirically. Based on responses from 400 of 965 invited hospitals, together with a parallel survey of 59 institutions conducted by the Center for Healthcare Quality Monitoring, the report found that every surveyed hospital collected some information on adverse events, but the methods of registration, monitoring and analysis varied so substantially between institutions that the resulting data were largely incomparable, and that the overall volume of reported events remained exceptionally low relative to the estimated scale of the problem (13).

Within the Polish legal framework, healthcare entities are assigned a broad range of responsibilities related to quality management and patient safety. Under Article 18 of the Act of 16 June 2023 on Quality in Healthcare and Patient Safety, healthcare organizations are required to identify and manage risks associated with adverse events, determine priority areas for quality improvement, establish criteria and methods for supervision, monitor and evaluate the quality and safety of healthcare services, maintain adverse event monitoring systems, provide staff training and collect information on patients' experiences and opinions.

Healthcare entities in Poland were required to implement an internal quality and safety management system by June 30, 2024. This obligation also stems from Article 18(1) of the Act on Quality in Healthcare and Patient Safety, under which an entity providing healthcare services—pursuant to a contract for the provision of publicly funded healthcare services concluded with the Fund in accordance with the Act on Healthcare Services—is required to maintain an internal system comprising principles, procedures, methods, and job descriptions designed to prevent the occurrence of adverse events.

An important component of the internal quality management system is Root Cause Analysis (RCA), which serves as a structured method for investigating adverse events. According to the Act, RCA involves collecting information about adverse events, mapping relevant data, identifying problems, analyzing contributing factors, determining root causes and formulating recommendations for changes in practice.

From an organizational perspective, the implementation of RCA may constitute a significant challenge for healthcare providers. Conducting a comprehensive RCA requires dedicated staff time, specialized training, access to relevant data and effective interdisciplinary cooperation.

The stages involving information mapping, identification of contributing factors and formulation of corrective actions are particularly resource-intensive.

The successful implementation of RCA therefore requires adequate human, organizational and financial resources (17, 18). Importantly, available evidence indicates ongoing efforts to strengthen organizational capacity in healthcare entities. In 2025, the Polish Patient Ombudsman provided training on patients' rights to nearly 6,000 healthcare professionals in 22 events and organized training and workshops for patient-rights representatives in healthcare entities. However, these activities do not directly demonstrate the availability of dedicated staff or financial resources for root cause analysis. Combined with the documented heterogeneity of adverse-event reporting systems, the continued use of paper-based reporting and low reporting volumes in many hospitals, they suggest that the organizational and information capacity required for systematic RCA is not yet uniformly developed across healthcare entities (14).

Within this framework, the manager of a healthcare entity is responsible for maintaining and supervising the internal quality and patient safety system.

The statutory responsibilities of the manager are broadly defined and, due to their scope, are frequently performed with the support of designated teams (19).

The most important tasks, in accordance with Article 19 of the Act of 16 June 2023 on Quality in Healthcare and Patient Safety (5, item 1692), include conducting root cause analyses, providing resources and information necessary for monitoring healthcare quality and safety, and developing documents specifying rules, procedures, methods and job descriptions.

Furthermore, according to Article 20 of the Act, patient opinions and experiences are assessed through an anonymized questionnaire administered after discharge from inpatient healthcare facilities or made available to other patients in a manner ensuring freedom of expression.

The implementation of an internal quality and safety management system also requires compliance with a number of formal organizational requirements.

These include a preliminary audit, risk assessment, the development of internal procedures, the establishment of organizational objectives, internal and certification audits, and the maintenance of an effective quality management system (20).

The operation of an internal system consists of implementing, maintaining and continuously improving the system on the basis of effectiveness assessments and the results of patient opinion surveys.

Potential benefits of implementing an internal quality and safety management system include increased awareness among medical personnel, improved patient satisfaction, enhanced organizational competitiveness and better preparation for accreditation processes.

### 3.3 Available empirical evidence and identified losses

In Poland, a system for reporting and monitoring adverse events was introduced primarily to enhance patient safety. The main objective behind collecting and analyzing information on adverse events is to ensure systemic learning from past experiences and to raise safety standards. Preventing adverse events is one area of healthcare—alongside initiatives such as health promotion and the reduction of behavioral risk factors (21)—where interventions can yield the greatest benefits for Polish patients. Data provided by healthcare entities in Poland indicate that the highest number of adverse events are reported in surgical wards; 30% of hospitals record reports concerning this department. Reports involving internal medicine wards occur in 20% of healthcare entities, while those concerning hospital emergency departments appear in 15.5% of hospitals. Information regarding gynecology and obstetrics wards was collected by 8% of the entities (13).

The fact that four hundred out of the nine hundred and sixty-five invited hospitals responded serves in itself as an indicator of the status of adverse event monitoring system implementation in Poland. “Information regarding adverse events is collected inadequately in the majority of hospitals, with 40% of facilities relying on paper-based methods for this purpose. Only a small percentage of hospitals possess a properly functioning adverse event registration system (…). Each of the surveyed hospitals in Poland collects information on adverse events; however, the methods used for their registration, monitoring, and analysis vary, often resulting in data that are incomparable and inconsistent. This lack of standardization hinders the identification of trends and the implementation of uniform solutions to enhance patient safety. Despite these challenges, the collected data reveal significant potential for implementing changes. The variations observed across hospitals provide a starting point for developing best practices that—with appropriate adaptation—could be implemented across all facilities. Introducing systematic qualitative and quantitative analyses could prove particularly valuable, as this would enable the identification of the root causes of events and the development of effective methods to prevent adverse events.” (…)

“The analysis reveals an exceptionally low number of adverse event reports in Polish hospitals. Regardless of hospital size—i.e., the number of beds and hospitalizations—reporting fewer than 50 adverse events certainly fails to reflect the actual situation within the facility (a figure reported by 60% of the surveyed entities). This points to underdeveloped reporting systems and a lack of awareness regarding adverse events among staff and healthcare providers. Furthermore, many hospitals collect reports on paper, which can hinder the reporting process. The analysis shows that adverse event reports are anonymous in 63% of facilities, although the stage at which anonymity is ensured varies by entity. Recommendations following event analysis are issued in over 76% of hospitals, while 57% monitor the effectiveness of the solutions implemented” (13).

## 4 Management of adverse events in different countries

At the national level, countries have adopted different legislative and institutional approaches to adverse event reporting and patient safety management.

In some Member States, only specific categories of adverse events are subject to mandatory reporting. This approach is applied, for example, in Poland and Hungary, where reporting obligations are linked primarily to the type of event.

By contrast, several countries, including the Czech Republic, Denmark, Ireland, Spain, Sweden and the United Kingdom, apply a broader concept of a “patient safety event,” allowing the reporting of virtually all incidents that may affect patient safety.

Norway represents a system in which reporting obligations are primarily determined by the severity of the event and focus on incidents resulting in serious patient harm. Patient safety in Norway is based on three legal pillars: patient rights, requirements for healthcare personnel and institutional responsibility. Instead of a single, comprehensive law, the system is governed by several complementary legal acts, with the principle of professional accountability playing a key role.

The Norwegian healthcare system emphasizes an open culture of learning from mistakes. The Helsetilsynet (Norwegian Health Supervision Authority) investigates serious adverse events, malpractice and patient complaints, while the Ukom (Norwegian Health Service Research Authority) investigates serious incidents and fatalities with the aim of improving the healthcare system and preventing similar events in the future^3^.

Denmark applies a mixed reporting model that combines both the type and severity of adverse events. The Danish healthcare system is widely regarded as one of the safest in the world and is based on several legal and organizational pillars. The Danish Patient Safety Authority (Styrelsen for Patientsikkerhed—STPS) is responsible for issuing professional authorizations, conducting risk-based supervision of medical facilities and developing guidelines concerning, among others, medication administration and medical documentation. Styrelsen for Patientklager is responsible for handling official patient complaints concerning medical errors and violations of patient rights^4^.

In Italy, patient safety is recognized as an integral element of the constitutional right to health guaranteed by Article 32 of the Constitution. The Gelli-Bianco Law (Legge n. 24/2017) establishes patient safety as a key component of the right to health and imposes a clinical risk management framework on all healthcare facilities. Italy's mixed system for reporting adverse events is characterized by the mandatory recording of the most serious incidents and the voluntary reporting of other, less severe events. Patient safety management is carried out within the framework of the Italian National Health Service^5^.

Germany represents a model that places particular emphasis on reporting near misses and developing systemic learning mechanisms. Patient safety in Germany is embedded within a decentralized and self-governing healthcare system.

The legal framework is based on four principal institutional pillars. The Patientenrechtegesetz (Patients' Rights Act) codifies patients' rights, including the right to information, access to medical records and informed consent. The CIRS (Critical Incident Reporting System) requires hospitals to implement internal quality management and incident reporting systems designed to prevent medical errors at the systemic level. SGB V (Book V of the Social Code) obliges healthcare institutions to implement continuous quality management, while the Joint Federal Committee (G-BA) establishes binding standards concerning treatment safety, hygiene and healthcare provision. Finally, the PDSG (Patient Data Protection Act) regulates the security and confidentiality of digital medical records within the national telematics infrastructure (22, 23).

Comparative evidence from outside Europe illustrates a similar diversity of institutional design. A comparison of incident reporting systems in Taiwan, Malaysia and Indonesia shows that even within the same region, countries differ substantially in the scope, mandatory character and organizational placement of their reporting mechanisms (24). Evaluations of hospital-level implementation in Indonesia point to comparable challenges regarding underreporting and limited use of collected data for systemic learning (25).

## 5 Incidence and impact of adverse events

Studies indicate that in high-income countries approximately one in ten patients experiences harm during hospitalization, whereas in low- and middle-income countries approximately 134 million adverse events occur annually, resulting in an estimated 2.6 million deaths (4). A landmark systematic review by de Vries et al. (26) estimated the median incidence of in-hospital adverse events at 9.2%, of which approximately 43.5% were judged preventable. Subsequent evidence has broadly confirmed and refined these figures. A 2018 scoping review of studies conducted after 2008 reported a median incidence of 10% (range 2.9–21.9%) and a median preventability of 51.2% (27). A larger meta-analysis covering 94 studies and nearly 590 million admissions found an incidence of 8.6 per 100 admissions (95% CI 8.3–8.9), with 52.6% of events classified as preventable (28). Another meta-analysis focusing specifically on preventable harm estimated a pooled prevalence of 6% (95% CI 5%−7%), of which 12% were severe or fatal (29).

The economic consequences of adverse events are substantial. According to the most recent OECD assessment, the direct cost of treating patients who have been harmed during care approaches 13% of total health expenditure in developed countries (approximately 8.7% when limited to potentially preventable harm). This corresponds to an estimated USD 606 billion annually across OECD countries, or just over 1% of their combined economic output (2, 30). Earlier OECD work had already indicated that roughly 15% of hospital activity and expenditure in high-income countries can be attributed to the additional care required after safety failures.

Polish data illustrate the same pattern at the micro level. An analysis of 100 compensation cases found that the average cost of secondary (restorative) treatment was EUR 1,433 compared with EUR 814 for the original (primary) treatment—almost a two-fold increase (31). These figures are based on court and compensation records from Poland and are expressed in nominal euros; they do not constitute a full economic evaluation with standardized cost categories, a defined reference population or adjustment to a common base year. The same study noted that compensation and damages related to adverse events increased by approximately 70% between 2014 and 2017. While this rise is consistent with broader European trends in litigation and payout levels, the estimate should be interpreted with caution because of the limited sample and the absence of detailed methodological breakdowns in the source.

Taken together, the updated epidemiological evidence and the OECD cost estimates confirm that adverse events continue to generate significant, largely avoidable human and financial burdens. Preventing even a fraction of these events therefore offers the potential for substantial reductions in both patient harm and healthcare expenditure.

## 6 Factors affecting the risk of adverse events

Adverse events in healthcare can be analyzed not only as a legal or organizational problem, but also as sudden health shocks affecting the economic situation and quality of life of patients and their households. In health economics, health is treated as a special form of human capital, and its sudden deterioration may lead to long-term consequences for an individual's professional activity, income and wellbeing (32). The concept of a health shock refers to an exogenous deterioration in health that disrupts household equilibrium and necessitates adaptive strategies, such as reducing labor supply or assuming informal caregiving responsibilities within the family (33, 34).

According to the WHO, factors that increase the risk of adverse events (35) include the lack of appropriate therapeutic training among doctors and nurses, patient misidentification, failure to recognize risks in diagnostic, nursing and therapeutic processes (36), excessive workload and fatigue among healthcare professionals, insufficient interpersonal communication, and problems related to the physical working environment, such as inadequate lighting, temperature and ventilation (37).

The European Commission defines an adverse event as harm arising during healthcare that is unrelated to the natural course of the disease or the patient's underlying condition (38, 71). Similarly, the WHO defines an adverse event as patient harm associated with healthcare rather than with disease progression (1). These definitions share a common objective of distinguishing preventable healthcare-related harm from the expected clinical course of illness.

Pokorski and Pokorska (39) additionally identify atypical reactions to medical procedures and staff fatigue as important risk factors. They emphasize that fatigue may result from financial pressures, multiple employment and staffing shortages and may consequently increase the probability of medical errors and adverse events (39).

The consequences of adverse events frequently extend beyond the healthcare system and impose substantial burdens on households, irrespective of formal legal liability. These burdens include both direct and indirect economic costs and a significant deterioration in health-related quality of life (40).

International strategic documents emphasize differences in the measurement of indirect and social costs associated with adverse events (2–4).

In particular, WHO documents place greater emphasis on the long-term consequences of adverse events for quality of life and wellbeing, whereas monitoring reports and healthcare statistics tend to focus primarily on systemic indicators and organizational performance.

This divergence highlights the need for a more comprehensive assessment of adverse events that simultaneously considers the macroeconomic consequences for healthcare systems and the microeconomic effects experienced by patients and their households.

## 7 Interventions for improving patient safety in healthcare systems

Examples of effective reporting and learning systems can be found in Australia and Canada.

In Australia, the Australian Open Disclosure Framework—Better Communication, a Better Way to Care was introduced by the Australian Commission on Safety and Quality in Health Care as a national standard for communicating adverse events to patients and their families (41). The framework promotes the development of reporting cultures, structured communication with patients and relatives, systematic documentation of disclosure processes and support for so-called “second victims,” namely healthcare professionals involved in adverse events (41, 42).

Similar principles can be observed in Canada. Canadian guidelines emphasize not only the disclosure of adverse events but also comprehensive support for patients and their families, including medical, emotional, psychological and practical assistance. The disclosure process requires healthcare professionals to communicate openly with patients, explain the circumstances of the event, discuss subsequent investigative steps and provide appropriate support.

Canadian guidelines also recognize the potential financial burden imposed on patients and allow reimbursement of selected expenses without constituting an admission of legal liability (43, 44).

Comparable disclosure programs have also been implemented in numerous healthcare organizations in the United States under frameworks such as Disclosure Programs, Communication and Resolution Programs (CRPs), and Disclosure, Apology and Offer (DA&O) programs (45).

Despite institutional differences, these programs share the common objective of promoting transparency, organizational learning and trust following adverse events.

The value of adverse event reporting extends beyond the documentation of individual incidents. According to international patient safety guidance, information obtained through reporting systems may be used to identify preventive measures, support organizational learning, facilitate the exchange of safety information across institutions, strengthen education and training activities, contribute to research and quality improvement initiatives, enhance accountability and promote open communication with patients and their families (4, 46, 47). Evaluation frameworks developed for patient safety incident reporting systems emphasize that data collection alone is insufficient; reporting systems must also include mechanisms that translate collected information into corrective action to be considered effective (48).

Looking ahead, the increasing use of artificial intelligence in diagnostic and decision-support systems raises new challenges for adverse event monitoring (49, 50). A future national register should therefore be designed to capture algorithmic errors and AI-related incidents. This need is reinforced by the documentation, logging and post-market monitoring requirements of the EU Artificial Intelligence Act (51), which align with the obligations already imposed by the Polish Act of 16 June 49 on Quality in Healthcare and Patient Safety.

The effectiveness of these systems depends on a supportive organizational culture in which healthcare professionals are encouraged to report incidents and where reporting is linked to learning and continuous improvement.

## 8 Discussion

The present study demonstrates that adverse events should be understood not only as clinical incidents or legal breaches but also as manifestations of broader systemic failures affecting healthcare governance, regulatory effectiveness and economic performance. The analysis shows that patient safety depends not solely on the existence of legal obligations but on the capacity of healthcare systems to generate reliable information, promote organizational learning and evaluate the effectiveness of regulatory interventions. These findings are consistent with the principles of the Global Patient Safety Action Plan 2021–2030, which identifies patient safety as a fundamental component of healthcare quality and emphasizes the need for comprehensive legal frameworks, transparent reporting systems and active patient participation in healthcare processes (4).

An important contribution of this study is the integration of legal analysis with institutional governance and health economics. While previous studies have frequently examined adverse events from clinical, organizational or legal perspectives, the present analysis demonstrates that reporting systems should also be understood as governance instruments that influence information flows, accountability and resource allocation within healthcare systems. This interdisciplinary perspective broadens the understanding of patient safety by linking regulatory design with wider questions of public policy and economic efficiency.

The findings further indicate that adverse events have consequences extending far beyond healthcare institutions. They reduce patients' health capital, generate productivity losses and impose substantial direct and indirect costs on patients, families and society. These observations support Grossman's concept of health capital (32) and correspond with evidence demonstrating that adverse events may function as health shocks requiring households to adapt through increased healthcare expenditure, reduced labor market participation and informal caregiving (33, 34, 52, 53). Consequently, the economic burden of adverse events should not be assessed exclusively from the perspective of healthcare providers but should also encompass the wider social costs transferred to households.

The study also highlights the importance of institutional conditions for effective patient safety regulation. Information asymmetry remains one of the defining characteristics of healthcare systems, limiting patients' ability to assess service quality and reducing the effectiveness of market mechanisms (54). Arrow's framework generates a concrete prediction: when the costs of disclosure fall primarily on the reporting party while the benefits accrue diffusely, systematic under-reporting is to be expected. The 2025 Patient Ombudsman survey supplies direct empirical support for this prediction-−60% of responding hospitals reported fewer than 50 adverse events regardless of size, 40% still relied on paper-based methods, and only a minority operated a fully functional electronic system.

## 9 Aligned interests vs. conflicts regarding the reporting of adverse events

In Poland, patients, doctors, hospital administrators, the National Health Fund (NFZ), insurers, and patient organizations all share an interest in improving treatment safety and eliminating systemic errors. Consequently, the common goal is to standardize procedures and take measures to reduce the incidence of adverse events; this translates into lower treatment costs—not only for the National Health Fund (the sole payer) but also directly for healthcare providers—and results in lower compensation payouts for insurers. Furthermore, a patient who avoids health impairment caused by an adverse event remains in much better health and functions as a more productive employee who does not need to take sick leave, thereby relieving the burden on the healthcare system.

Beyond these points of intersection, a clear conflict exists between transparency and accountability on the one hand, and legal and financial security on the other. Patients value transparency in the reporting of adverse events—seeking compensation or redress for harm suffered—and prefer to choose medical facilities with the lowest incidence of such events. Conversely, doctors are reluctant to disclose adverse events in the absence of a “no-fault” system that would exempt them from criminal liability for unintentional errors; furthermore, they often fear that the medical facility might lose prestige or that they themselves could face legal action. The failure to report adverse events stems from concerns regarding legal liability, shared by both medical professionals and the administrators of medical facilities. This conclusion is supported by a study covering 15% of Polish hospitals, which indicates that requests from law enforcement agencies for information on adverse events can also deter the reporting of such incidents (13).

Financial implications also frequently hinder the monitoring of adverse events. Healthcare providers often delegate the responsibility for tracking these events to specific groups—typically assigning the role to a director, an infection control nurse, or an infection control team—or to an individual, such as a member of the management team. Directors of healthcare facilities argue that analyzing these events entails increasing staffing levels and raising costs for staff training on quality and safety—covering both new hires and the professional development of existing employees. Conversely, the National Health Fund expects a marked improvement in the quality of healthcare services and reporting standards as part of its contracts with providers. Patient organizations also expect prompt insurance payouts, while insurers frequently raise premiums for healthcare providers based on reported adverse events. Such a palpable conflict can lead to numerous irregularities and systemic abuses within the healthcare sector, potentially resulting not only in a failure to monitor adverse events but, more importantly, in their deliberate concealment.

## 10 Operationalization and estimation of labor-resource costs associated with Article 18

The annual labor-resource cost associated with the obligations arising from Article 18 of the Act of 16 June 2023 on Quality in Healthcare and Patient Safety was estimated. The estimand is not the total cost of operating a quality system, nor the full transaction cost of regulation. It is a scenario-based annual cost of staff time devoted to running the internal system, including the monitoring of adverse events, their analysis and root-cause analysis (RCA).

The cost was calculated according to the following formula (Equation 1):


$$\begin{array}{l} C=N\times f\times k\backslash n \end{array}$$ (1)

- *N* denotes the number of entities included in the population,
- *f* denotes the average full-time equivalent (FTE) allocated to system-related tasks in a single entity,
- *k* denotes the annual full employer cost of one FTE.

As the baseline value of *N*, 18 042 entities were adopted. This figure appears in the AOTMiT report WT.543.4.2025 (Table 1), which was compiled on the basis of National Health Fund data as of 19 May 2025 (69).

**Table 1:** Comparison of adverse event reporting systems in selected European countries.

Country	Legal basis	Competent authority	Mandatory/voluntary reporting	National register	Legal protection for the reporter	Destination of collected data
Poland	Act of 16 June 2023 on Quality in Healthcare and Patient Safety (Arts. 18–20)	Healthcare entity manager	Mandatory (internal systems in entities with NFZ contracts)	None	Limited (partial anonymity; no clear protection against liability)	Internal analysis and root cause analysis at entity level only
Norway	Helsetilsynsloven + Act on the Norwegian Healthcare Investigation Board (Ukom)	Helsetilsynet, Ukom	Mandatory (based on severity—death or very serious injury)	Partial (notification system; national register under consideration)	Strong learning-oriented protection; reports used for improvement, not punishment	Investigation of causes, improvement reports, systemic learning and prevention
Denmark	Lov om patientsikkerhed (2003, as amended) + Sundhedsloven (68)	Danish Patient Safety Authority (STPS)	Mandatory (serious/fatal consequences + learning-relevant events); aggregated reporting possible	Yes—Danish Patient Safety Database (DPSD)	Sanction-free (no-blame) reporting; protection against liability for unintentional errors	National analysis, feedback to staff, systemic learning, annual reports
Italy	Legge 8 marzo 2017, n. 24 (Gelli-Bianco)	Regional Centers for Clinical Risk Management + National Observatory on Good Practices	Mixed (mandatory for most serious/sentinel events; voluntary for others)	Partial (data transmitted to the national observatory)	Encouragement of reporting within clinical risk management; reduced criminal liability when guidelines followed	Clinical risk management, annual reports, national good practices
Germany	Patientenrechtegesetz + SGB V + G-BA regulations + CIRS framework	Joint Federal Committee (G-BA), hospital CIRS systems	Mainly voluntary (near misses and events), with mandatory quality elements	No uniform national register (decentralized CIRS systems)	Strong emphasis on learning culture; reporting of near misses without fear of sanctions	Systemic learning, quality improvement, G-BA standards

In the present analysis the figure of 18 042 is treated as an operational approximation of the population of providers holding contracts with the National Health Fund, rather than as a definitive legal census of every entity formally covered by Article 18.

Because the available evidence does not contain direct observations of the time actually spent on these tasks, the FTE share was treated as a scenario parameter rather than an empirically measured value. Three variants were adopted: low (0.25 FTE per entity), baseline (0.50 FTE per entity), high (1.00 FTE per entity).

For the annual full employer cost of one FTE, three scenario values were applied: PLN 110 000, PLN 125 000 and PLN 140 000. These figures were derived from median gross salaries of quality specialists reported in the Ogólnopolskie Badanie Wynagrodzeń (55) and adjusted by a standard employer cost multiplier of approximately 1.22.

The estimate deliberately excludes: costs of software, IT implementation and system maintenance, training costs, audit costs, data-protection costs, opportunity costs of staff time.

For the baseline scenario, the estimated annual value of labor resources is calculated as follows:

18,042 entities × 0.50 FTE × PLN 125,000 = PLN 1,127,625,000 per year, which corresponds to approximately EUR 259.6 million per year.

For international comparability, all monetary values in the results are reported in euros (EUR). The conversion was performed using the NBP average exchange rate of EUR 1 = PLN 4.3435, applicable on 16 September 2026. The complete set of scenario results is presented in Table 2.

**Table 2:** Scenario-based estimates of annual labor-resource costs associated with the internal quality and adverse event monitoring system under Article 18.

Scenario	FTE per entity	Annual employer cost per FTE (PLN)	Annual employer cost per FTE (EUR)	Estimated annual labor cost (EUR)
Low	0.25	110,000	25,325	EUR 114.23 million
Low	0.25	125,000	28,779	EUR 129.81 million
Low	0.25	140,000	32,232	EUR 145.38 million
Baseline	0.50	110,000	25,325	EUR 228.46 million
**Baseline**	**0.50**	**125,000**	**28,779**	**EUR 259.61 million**
Baseline	0.50	140,000	32,232	EUR 290.77 million
High	1.00	110,000	25,325	EUR 456.92 million
High	1.00	125,000	28,779	EUR 519.22 million
High	1.00	140,000	32,232	EUR 581.53 million

Source: Authors' own calculations. The number of entities (18,042) is based on the AOTMiT report WT.543.4.2025 and reflects National Health Fund data as of 19 May 2025. The assumed FTE allocation per entity and annual employer cost per FTE are modeling assumptions. PLN amounts were converted into EUR using the NBP average exchange rate of EUR 1 = PLN 4.3435 as of 16 September 2026. Bold values indicate the baseline scenario assumptions and the resulting estimated annual labor cost.

The sensitivity analysis illustrates the extent to which the estimate depends on the assumed labor intensity and unit cost. With an allocation of 0.25 FTE per entity, the estimated annual labor-resource value ranges from approximately EUR 114.2 million to EUR 145.4 million, depending on the assumed annual employer cost per FTE. Under the baseline allocation of 0.50 FTE per entity, the corresponding estimates range from EUR 228.5 million to EUR 290.8 million. Under the high-intensity scenario of 1.00 FTE per entity, the estimated annual value ranges from approximately EUR 456.9 million to EUR 581.5 million. The estimates highlight the potential for duplicated effort that a national infrastructure could reduce.

From the perspective of economic theories of regulation, public interventions should therefore be evaluated not only according to formal legal compliance but also in terms of their effectiveness, efficiency and capacity to reduce information failures (56, 57).

Grossman's health-capital model supplies the micro-economic counterpart (32). An adverse event that produces lasting impairment reduces an individual's stock of health capital, lowering subsequent labor productivity and increasing household demand for informal care. While precise national quantification is constrained by the lack of linked administrative data, the two-fold difference between primary and secondary treatment costs reported by Leśniak et al. (31) already indicates the magnitude of the direct medical component of such shocks.

This interpretation is consistent with international recommendations. Both the OECD and the WHO emphasize that effective patient safety systems require coherent institutional frameworks integrating measurement, reporting, organizational learning and continuous quality improvement (2, 4). Similarly, WHO guidance on adverse event reporting highlights the importance of organizational cultures based on trust, transparency and learning, supported by the principles of no-blame and just culture (46, 58). These approaches are intended to encourage incident reporting while maintaining accountability for reckless or intentional misconduct.

The Polish case illustrates both the progress achieved and the challenges that remain. The introduction of mandatory internal adverse event monitoring systems constitutes an important regulatory development that strengthens organizational responsibility for patient safety. However, the present analysis indicates that a decentralized reporting model provides limited opportunities for nationwide organizational learning, comparative evaluation and evidence-based policymaking. From the perspective of institutional economics, the absence of an integrated national reporting system may increase transaction costs associated with obtaining and exchanging information while perpetuating information asymmetries across the healthcare system (54, 59).

Regulatory Impact Assessment principles emphasize that public interventions should be assessed on the basis of empirical evidence obtained through both ex ante and ex post evaluations (60, 61). Without integrated and comparable national data, it remains difficult to determine whether the costs associated with adverse event monitoring systems are justified by measurable improvements in patient safety. This limitation also constrains reliable cost-benefit analyses of patient safety policies (62–64).

Nevertheless, more comprehensive empirical research is required to quantify these relationships and assess the cost-effectiveness of alternative regulatory models (31, 65, 66).

From a public policy perspective, the present findings support further development of integrated adverse event reporting systems, including consideration of a national adverse event register. Such a solution could strengthen organizational learning, improve transparency, facilitate evidence-based policymaking and provide a more comprehensive understanding of the economic and social consequences of adverse events. More broadly, the study demonstrates that integrating legal analysis with institutional governance and health economics provides a stronger foundation for designing patient safety regulations capable of improving both healthcare quality and the efficient allocation of public resources.

## 11 Conclusion

This study demonstrates that patient safety should be understood not only as a clinical or legal issue but also as a matter of healthcare governance and health economics. By integrating legal analysis with institutional and economic perspectives, the article shows that adverse event reporting systems perform functions that extend beyond documenting individual incidents. They constitute mechanisms for organizational learning, regulatory evaluation and evidence-based policymaking, while simultaneously influencing the allocation of healthcare resources and the distribution of the social costs associated with preventable patient harm.

The analysis indicates that the effectiveness of patient safety regulation depends not only on the existence of legal obligations but also on the quality of the institutional infrastructure supporting their implementation. International experience demonstrates that effective reporting systems are characterized by transparency, organizational learning and the systematic use of information for quality improvement. Against this background, the Polish model represents an important step toward strengthening patient safety governance through mandatory internal monitoring systems. However, its predominantly decentralized structure still limits nationwide learning and comparative evaluation.

From a policy perspective, strengthening patient safety requires not only maintaining internal reporting obligations but also improving information governance at the national level. The development of integrated reporting mechanisms capable of supporting evidence-based regulatory evaluation would facilitate more effective patient safety policies, improve organizational learning and provide a stronger basis for assessing the economic consequences of adverse events. Such solutions could contribute to both better health outcomes and more efficient allocation of healthcare resources.

This study has several limitations. It is based primarily on doctrinal legal analysis supported by institutional and health economics literature and does not include empirical analyses of adverse event databases or quantitative assessments of the costs and benefits of specific regulatory interventions. Consequently, the conclusions concerning the potential economic effects of reporting systems should be interpreted as theoretically grounded rather than empirically verified. A further limitation concerns language accessibility: a substantial part of the doctrinal and institutional analysis relies on Polish-language sources (e.g., 20, 21, 24, 36) that are not available in English and cannot be independently verified by international readers. Where feasible, key claims drawn from these sources have been paraphrased in English within the text; nevertheless, readers without Polish-language proficiency should treat these claims as reported rather than independently verifiable. Future research should therefore combine legal analysis with empirical investigations of adverse event reporting systems, comparative international studies and economic evaluations examining the effectiveness and cost-effectiveness of different regulatory models.

## Footnotes

1 Act of 23 April 1964 – Civil Code, Journal of Laws of 2026, item 795.

2 Regulation of the Minister of Health of 10 June 2024 on the method of determining the amount of the compensation benefit for infection with a biological pathogen, bodily injury, impairment of health, or the death of a patient, Journal of Laws of 2024, item 883.

3 Lov om Statens undersøkelseskommisjon for helse-og omsorgstjenesten, LOV-2025-06-20-74.

4 Lov om patientsikkerhed i sundhedsv6senet (lov nr. 429 af 10. juni 2003) var Danmarks og verdens f8rste nationale lov om patientsikkerhed.

5 LEGGE 8 marzo 2017, n. 24 Disposizioni in materia di sicurezza delle cure e della persona assistita, nonche' in materia di responsabilita' professionale degli esercenti le professioni sanitarie. (17G00041) (GU Serie Generale n.64 del 17-03-2017).
